# Support, Health, Activities, Resources, & Education (SHARE): New Program Innovations for Early-Stage Families

**DOI:** 10.1093/geroni/igaa057.2133

**Published:** 2020-12-16

**Authors:** Carol Whitlatch, Steven Zarit, Steven Zarit

## Abstract

Persons living with a recent diagnosis of dementia experience great uncertainty and stress as they and their families try to adjust to the new reality of their lives and futures. One fruitful strategy for intervening with these families is to include both the person living with dementia and their family care partner in the program. Although dyadic approaches are rare among early-stage programs, promising examples exist. The SHARE Program (Support, Health, Activities, Resources, and Education) is one exception where dyadic materials address: 1) current and long- term needs of care partners, and 2) how the family can develop a realistic plan of care based on their care values and preferences. This symposium describes the development and positive outcomes of the original SHARE intervention and the promising adaptations that expand how and to whom the intervention is delivered. Presentations explore: 1) the original SHARE for Dementia program and strategies for expanding its reach into chronic conditions populations (Orsulic-Jeras & Whitlatch), 2) a group version translated into Spanish (“EPIC: Early-stage Partners in Care,” Dr. Coon), and 3) the development of a remote needs assessment and unobtrusive in-home monitoring technology platform that guides care planning and helps to maintain independence (“SHARE-sense,” Dr. Miller). Discussion will focus on the challenges, unique solutions, and positive outcomes when adapting SHARE to different settings and populations (Dr. Zarit).

